# Supplementary material for: Diet-Related Attitudes, Beliefs, and Well-Being in Adolescents with a Vegetarian Lifestyle
Source: Healthcare (Basel). 2023 Nov 2;11(21):2885. doi: 10.3390/healthcare11212885 (PMC10650094; doi:10.3390/healthcare11212885)
Supplement: Supplementary file 1 [file healthcare-11-02885-s001.zip › healthcare-2561763-supplementary.pdf]

Prof.ssa Loredana Benedetto

Dipartimento di Medicina Clinica e Sperimentale
Corso di Laurea Magistrale in Psicologia
UNIVERSITÀ DEGLI STUDI DI MESSINA

Stiamo conducendo uno studio sulle scelte e sulle abitudini alimentari dei ragazzi della tua età. Per questo motivo ti proponiamo di rispondere **in forma anonima** alle domande qui sotto elencate. Ciò che vogliamo conoscere sono le tue opinioni e esperienze: rispondi quindi in maniera sincera, perché **non ci sono risposte "giuste" o "sbagliate"**!

Per favore, cerca di rispondere a tutte le domande. *Grazie!*

1. I MIEI ATTEGGIAMENTI VERSO L'ALIMENTAZIONE

Quanto TU sei d'accordo con ciascuna di queste affermazioni?	Per nulla d'accordo	Un po' d'accordo	Non so	Molto d'accordo	Totalmente d'accordo
1. La dieta è importante nella prevenzione di malattie e disagi					
2. Non ho bisogno di apportare cambiamenti nel cibo che mangio perché esso è già abbastanza sano					
3. Di solito non penso agli aspetti nutrizionali dei tipi di cibo che mangio					
4. Frequentemente cerco informazioni su come mangiare in modo salutare					

2. LE MIE OPINIONI SUL CONSUMO DELLA CARNE

1. La carne è importante per crescere forti					
2. Preferisco mangiare carne rossa rispetto a frutta o verdura					
3. Penso che mangiare carne sia disgustoso					
4. Niente soddisfa il mio appetito come una spessa bistecca gustosa					
5. La carne come manzo o agnello non è sana da mangiare					
6. La carne rossa (come manzo o agnello) fa ingrassare					
7. La carne è necessaria nella dieta delle persone adulte					
8. La carne è necessaria nelle diete dei bambini					
9. La carne causa problemi di salute (come cancro e problemi cardiaci)					
10. La carne rossa è la fonte di ferro meglio assorbita nella dieta					
11. I non-vegetariani sono più in salute rispetto ai vegetariani					
12. La carne rossa è molto costosa					
13. Quando mangio fuori per festeggiare una ricorrenza o un'occasione sociale, di solito mangio qualche tipo di carne rossa					
14. La carne è il cibo che gli uomini sono più propensi a mangiare					
15. La produzione di carne è crudele per gli animali					
16. Gli uomini non hanno diritto di uccidere gli animali per nutrirsi					
17. I vegetariani sono hippy o stravaganti					

3. LA MIA DIETA

Quanto spesso mangi i seguenti cibi?<br>**(*Segna la tua risposta accanto a ciascun cibo*)**	Mai	Raramente	1-3 volte al mese	1-4 volte la settimana	Giornalmente o quasi tutti i giorni
1. Carne rossa (ad es. manzo, agnello, vitello)					
2. Carne bianca (ad es. pollo, maiale)					
3. Pesce e frutti del mare (ad es. cozze, vongole)					
5. Latticini (ad es. latte, formaggi, burro)					

4. IL VEGETARIANISMO

4.1 Hai sentito parlare di vegetarianismo? ***(Cerchia la TUA risposta)***

1. No
2. Si

4.2 Sei interessato al vegetarianismo?

1. No, non mi interessa
2. Non so
3. Un po' interessato
4. Molto interessato

4.3 Nell'ultimo mese, hai pensato di cambiare le tue abitudini adottando una dieta vegetariana?

1. No
2. Qualche volta
3. Abbastanza spesso
4. Molto a lungo
5. Io sono vegetariano/a

4.4 In passato, sei mai stato vegetariano?

1. No
2. Sì **Se hai risposto Si, per quanto tempo sei stato vegetariano? Per ______ anno/i e/o ______ mese/i**

4.5 Sei vegetariano ora?

1. No
2. Sì
3. Normalmente penso a me stesso come a un semi-vegetariano

Se hai risposto SI o SEMI_VEGETARIANO, da quanto tempo sei vegetariano o semi-vegetariano?
Da ______ anno/i e/o ______ mese/i

4.6 Durante i prossimi tre mesi, quanto è probabile che cambierai la tua dieta per diventare vegetariano?

1. Non è per nulla probabile
2. Un po' probabile
3. Probabile
4. Molto probabile
5. Non so
6. Sono vegetariano

4.7 Hai qualche membro in famiglia che è vegetariano? ***(Se necessario, cerchia più di un numero)***

1. Nessuno
2. Padre
3. Madre
4. Fratello/sorella
5. Fidanzato/fidanzata

4.8 Hai qualche amico che è vegetariano?

1. No, nessuno
2. Sì, qualcuno **Se hai risposto SI, indica quanti tuoi amici sono vegetariani: _______amico/i vegetariano/i**

5. DIFFICOLTA' CON LE DIETE VEGETARIANE

**Essere vegetariano potrebbe essere difficoltoso per me perché…** ***(Per favore, segna quanto sei d'accordo con ciascuna affermazione)***	Per nulla d'accordo ☐	Un po' d'accordo ☐	Non so ☐	Molto d'accordo ☐	Totalmente d'accordo ☐
1. I miei amici mangiano carne	☐	☐	☐	☐	☐
2. Nella mia famiglia si mangia carne	☐	☐	☐	☐	☐
3. A me piace mangiare carne	☐	☐	☐	☐	☐
4. Mi sentirei diverso dagli altri	☐	☐	☐	☐	☐

5. Non c'è abbastanza ferro nelle diete vegetariane	☐	☐	☐	☐	☐
6. La dieta vegetariana causa carenze di vitamine (B, D ecc.)	☐	☐	☐	☐	☐
7. Non ci sono abbastanza proteine nelle diete vegetariane	☐	☐	☐	☐	☐
8. Sono preoccupato/a per la mia salute (problemi diversi da carenza di ferro, vitamine o di proteine)	☐	☐	☐	☐	☐
9. I miei familiari decidono della maggior parte del cibo che mangio	☐	☐	☐	☐	☐
10. La mia famiglia non vorrebbe mangiare cibo vegetariano	☐	☐	☐	☐	☐
11. Preparare cibo vegetariano richiede molto tempo	☐	☐	☐	☐	☐
12. Non voglio mangiare cibi poco comuni o strani	☐	☐	☐	☐	☐
13. Le pietanze vegetariane a casa mia non si consumano	☐	☐	☐	☐	☐
12. Non ho abbastanza forza di volontà	☐	☐	☐	☐	☐
13. Penso che gli uomini siano fatti per mangiare carne	☐	☐	☐	☐	☐
14. Non so cosa mangiare al posto della carne	☐	☐	☐	☐	☐
15. Non voglio cambiare le mie abitudini alimentari	☐	☐	☐	☐	☐
16. C'è una scelta troppa limitata quando mangio fuori	☐	☐	☐	☐	☐
17. Le diete vegetariane non riempiono abbastanza	☐	☐	☐	☐	☐
18. Le diete vegetariane sono noiose	☐	☐	☐	☐	☐
19. Non voglio che le persone mi etichettino negativamente (ad es. come una persona strana)	☐	☐	☐	☐	☐
20. Non avrei abbastanza energie o forza dal cibo	☐	☐	☐	☐	☐
21. Ho bisogno di più informazioni sulle diete vegetariane	☐	☐	☐	☐	☐

ALTRA DIFFICOLTA' (PER FAVORE SPECIFICA)____

Per favore, cerchia il numero corrispondente alla PRINCIPALE DIFFICOLTA' per TE con la dieta vegetariana

**Cosa pensi del tuo peso?**	Mi piacerebbe aumentare di parecchi chili	Mi piacerebbe aumentare di 2-3 chili	Il mio peso è giusto	Mi piacerebbe perdere 2-3 chili	Mi piacerebbe peredere parecchi chili

6. BENEFICI DELLE DIETE VEGETARIANE

**Io credo che una dieta vegetariana potrebbe <u>aiutare</u> a…**	Per nulla d'accordo ☐	Un po' d'accordo ☐	Non so ☐	Molto d'accordo ☐	Totalmente d'accordo ☐
1. Controllare il mio peso	☐	☐	☐	☐	☐
2. Prevenire le malattie in generale (ad esempio, malattie cardiache, cancro)	☐	☐	☐	☐	☐
3. Ridurre l'assunzione di grassi saturi nella mia dieta	☐	☐	☐	☐	☐
4. Vivere più a lungo	☐	☐	☐	☐	☐
5. Mangiare una più grande varietà di cibi interessanti	☐	☐	☐	☐	☐
6. Avere energie in abbondanza	☐	☐	☐	☐	☐
7. Avere una migliore qualità di vita	☐	☐	☐	☐	☐
8. Stare bene	☐	☐	☐	☐	☐
9. Essere meno aggressivo	☐	☐	☐	☐	☐
10. Essere più soddisfatto/a di me stesso/a	☐	☐	☐	☐	☐
11. Risparmiare soldi	☐	☐	☐	☐	☐
12. Avere una dieta più gustosa	☐	☐	☐	☐	☐
13. Soddisfare i miei bisogni religiosi e/o spirituali	☐	☐	☐	☐	☐
14. Essere in forma	☐	☐	☐	☐	☐
15. Mangiare più frutta e verdura	☐	☐	☐	☐	☐
16. Essere più in salute diminuendo il consumo di sostanze chimiche (come steroidi e antibiotici) contenute nella carne	☐	☐	☐	☐	☐

17. Aumentare il controllo sulla mia salute	▫	☐	☐	☐	☐
18. Ridurre le possibilità di intossicazione alimentare	▫	☐	☐	☐	☐

**Credo che il vegetarianismo possa aiutare …**	Per nulla d'accordo ▫	Un po' d'accordo ☐	Non so ☐	Molto d'accordo ☐	Totalmente d'accordo ☐
19. A ridurre l'inquinamento ambientale	▫	☐	☐	☐	☐
20. A diminuire la fame nel Terzo Mondo	▫	☐	☐	☐	☐
21. Il benessere/la tutela degli animali	▫	☐	☐	☐	☐
22. Una più efficiente produzione di cibo	▫	☐	☐	☐	☐
23. A educare al rispetto per le differenze	▫	☐	☐	☐	☐
24. Creare un mondo più pacifico	▫	☐	☐	☐	☐

ALTRO BENEFICIO (PER FAVORE SPECIFICA)____

Quale pensi sia il PRINCIPALE BENEFICIO della dieta vegetariana? Per favore, cerchia il numero corrispondente.

8. AUTONOMIA

**Quanto è importante per te…**	Per nulla importante ▫	Poco importante ☐	Abbastanza importante ☐	Molto importante ☐
1. Essere in grado di decidere, in modo indipendente dagli adulti, come impiegare il tempo libero	▫	☐	☐	☐
2.Essere libero di usare i soldi che hai nel modo che desideri	▫	☐	☐	☐
3. Scegliere da solo/a cosa mangiare	▫	☐	☐	☐
4. Scegliere da solo/a i tuoi vestiti	▫	☐	☐	☐

**Negli ultimi sei mesi, quanto ti sei sentito…**	Per nulla d'accordo ▫	Poco d'accordo ☐	Abbastanza ☐	Molto d'accordo ☐
1. Giù di morale per certi eventi	▫	☐	☐	☐
2. Senza speranza per il futuro	▫	☐	☐	☐
3. Preoccupato/a eccessivamente per piccole cose	▫	☐	☐	☐
4. Depresso/a circa la vita in generale	▫	☐	☐	☐
5. Solo/a	▫	☐	☐	☐

**Quanto sono severi i tuoi genitori con te?**	Per nulla	Poco	Abbastanza	Molto

9. I TUOI VALORI PERSONALI

Un fattore importante per comprendere le scelte alimentari delle persone è la loro visione del mondo, di conseguenza questa sezione del questionario riguarda i valori personali. Quanto sono importanti i seguenti principi guida nella TUA_ vita?

**Quanto sono importanti i seguenti principi guida nella TUA vita?** (***Per favore, esprimi la TUA risposta per ciascuno dei seguenti valori***)	Contrario ai miei valori	Non importante	Abbastanza importante	Molto importante
1. Giustizia (pari opportunità per tutti gli esseri viventi)	1	2	3	4
2. Armonia interiore (essere in pace con me stesso/a)	1	2	3	4
3. Influenza sociale (influenza sul comportamento degli altri)	1	2	3	4
4. Ordine sociale (sicurezza della società)	1	2	3	4
5. Una vita eccitante (esperienze stimolanti)	1	2	3	4

6. Ricchezza (possedere beni materiali, soldi)	1	2	3	4
7. Auto-disciplina (avere autocontrollo)	1	2	3	4
8. Sicurezza familiare (sicurezza dei propri cari)	1	2	3	4
9. Unione con la natura (sentirsi in armonia e adattarsi alla natura)	1	2	3	4
10. Autorità (il diritto di essere un leader o comandare)	1	2	3	4
11. Un vita varia (piena di sfide, novità e cambiamento)	1	2	3	4
12. Un mondo di bellezza (bellezza della natura e delle arti)	1	2	3	4
13. Proteggere l'ambiente (preservare la natura)	1	2	3	4
14. Rispetto per i genitori e gli anziani (mostrare considerazione per genitori/anziani)	1	2	3	4
15. Godersi la vita (cibo, sesso, tempo libero ecc.)	1	2	3	4
16. Devozione (rispettare le credenze e la fede religiosa)	1	2	3	4
17. Salute (non ammalarsi nel fisico o nella mente)	1	2	3	4
18. Rispetto per la tradizione (mantenere le usanze consolidate nel tempo)	1	2	3	4
19. Preservare la mia immagine pubblica (proteggere la mia "faccia")	1	2	3	4
20. Indipendenza (autonomia, autosufficienza)	1	2	3	4
21. Aiutare (impegnarsi per il benessere degli altri)	1	2	3	4
22. Moderatezza (evitare gli eccessi nei sentimenti e nelle azioni)	1	2	3	4
23. Successo (raggiungere obiettivi)	1	2	3	4

Quando hai finito, per favore cerchia il numero del valore che è più importante per te.

Infine, vorremmo chiederti qualche informazione su di te per scopi statistici.

<table>
<tr><td colspan="2">10. Qual è il tuo genere? 1. MASCHILE 2. FEMMINILE</td><td>11. Qual è la tua età? ________ (anni)</td></tr>
<tr><td>12. Qual è il livello di istruzione di tuo padre?
1. LICENZA ELEMENTARE
2. LICENZA MEDIA
3. DIPLOMA DI MATURITA'
4. LAUREA
5. SPECIALIZZAZIONE/MASTER UNIVERSITARIO</td><td>13. Qual è il livello di istruzione di tua madre?
1. LICENZA ELEMENTARE
2. LICENZA MEDIA
3. DIPLOMA DI MATURITA'
4. DIPLOMA DI LAUREA
5. SPECIALIZZAZIONE/MASTER UNIVERSITARIO</td><td>14. Qual è la tua religione?
1. CRISTIANA
2. MUSULMANA
3. BUDDISTA
4. EBRAICA
5. NESSUNA RELIGIONE (AGNOSTICO O ATEO)
6. ALTRA (per favore, specifica):</td></tr>
<tr><td>15. Qual è il tuo peso?
________ (Kg)</td><td>16. Qual è la tua altezza?
________ (cm)</td><td>17. Qual è il tuo orientamento sessuale?
1. OMOSESSUALE
2. BISESSUALE
3. ETEROSESSUALE
4. ASESSUALE</td></tr>
</table>

Grazie per la tua partecipazione a questa ricerca
